# Der Schwangerschaftsabbruch aus multidisziplinärer Perspektive

**DOI:** 10.1007/s00103-024-03996-1

**Published:** 2025-01-14

**Authors:** Matthias David, Joseph Kuhn, Anke-Christine Saß

**Affiliations:** 1 Klinik für Gynäkologie, Charité – Universitätsmedizin Berlin, Campus Virchow Klinikum, Augustenburger Platz 1, 13353 Berlin, Deutschland; 2 Pettenkofer School of Public Health München, c/o Bayerisches Landesamt für Gesundheit und Lebensmittelsicherheit, Veterinärstr. 2, 85764 Oberschleißheim, Deutschland; 3 Abteilung für Epidemiologie und Gesundheitsmonitoring, Robert Koch-Institut, Gerichtstr. 27, 13347 Berlin, Deutschland

Das Thema Schwangerschaftsabbruch steht politisch derzeit in Deutschland wieder auf der Agenda. 2022 wurde das sog. Werbeverbot für Schwangerschaftsabbrüche (§ 219 a StGB) abgeschafft, das einer befürchteten Kommerzialisierung von Schwangerschaftsabbrüchen entgegenwirken sollte, von dem aber vermutet wurde, dass es zugleich versorgungsrelevante Informationen für Frauen erheblich eingeschränkt hatte. Ende 2024 wurde mit einer Änderung des Schwangerschaftskonfliktgesetzes ein Verbot der sog. Gehsteigbelästigungen explizit im Recht verankert. Es soll Schwangere, die eine Beratungsstelle aufsuchen, davor schützen, vor den Beratungsstellen von Abtreibungsgegnerinnen und -gegnern angesprochen und auch bedrängt zu werden. Diskutiert werden Vorschläge, den Schwangerschaftsabbruch, der seit 150 Jahren nach § 218 StGB als Straftatbestand gilt, auch wenn er inzwischen unter bestimmten Bedingungen zwar rechtswidrig, aber straffrei ist, als Straftatbestand grundsätzlich zu streichen. Eine von der Bundesregierung eingesetzte Kommission aus Expertinnen und Experten hat dies im April 2024 empfohlen. Politisch ist das jedoch strittig, eine Neuregelung des Strafrechts ist zumindest kurzfristig nicht in Sicht. Auch die Bevölkerung zeigt sich in Umfragen uneins. Verglichen mit überwiegend katholischen Ländern Südeuropas steht sie einer Streichung des Straftatbestandes offener gegenüber, verglichen mit den nordeuropäischen Ländern ist sie reservierter.

Mit diesen politischen Debatten eng verbunden sind statistische, medizinische, soziologische, psychologische und ethische Fragestellungen, die Gegenstand des vorliegenden Themenheftes sind. Wie in anderen politisch umstrittenen Themenbereichen sind auch hier die wissenschaftlichen Perspektiven nicht immer frei von wertebasierten Positionen, aber vermutlich ist das Ideal einer Wissenschaft ohne Werturteile ohnehin nur schwer erreichbar, vielleicht auch gar nicht immer erstrebenswert. Dessen ungeachtet ist es notwendig, die Daten, auf die sich wertebasierte Positionen stützen, wissenschaftlichen Standards entsprechend zu erheben, auszuwerten und zu interpretieren, sodass Wissenschaft und Politik nicht in unzulässiger Weise vermischt werden. Die vielzitierte Warnung des österreichischen Soziologen Alexander Bogner vor einer „Epistemisierung des Politischen“, dem Verstecken von Interessenskonflikten und standortgebundenen Interpretationen hinter wissenschaftlichen Aussagen, muss auch hier gelten.

Im Jahr 2023 verzeichnete das Statistische Bundesamt 106.218 Schwangerschaftsabbrüche in Deutschland, etwas mehr als im Vorjahr. Die ganz überwiegende Anzahl der Schwangerschaftsabbrüche findet ambulant statt, entweder in gynäkologischen Praxen oder in ambulanten OP-Zentren. Dabei sind flächendeckende und wohnortnahe Versorgungsangebote für Frauen in dieser schwierigen Situation wichtig.

Über die Ursachen für Schwangerschaftsabbrüche gibt die Statistik nur eingeschränkt Auskunft. Fast alle Schwangerschaftsabbrüche – 95 % – sind auf der Grundlage der Beratungsregelung erfolgt, eine der Formen, bei der der vom Grundsatz her strafbewehrte Abbruch nach § 218a StGB straflos bleibt. Den anderen 5 % lag zumeist eine medizinische Indikation zugrunde, in wenigen Fällen eine kriminologische. Eine amtliche Schwangerschaftsabbruchstatistik ist übrigens keine Selbstverständlichkeit: Österreich hat so etwas beispielsweise nicht.

Über die individuellen und sozialen Hintergründe der Schwangerschaftsabbrüche auf Grundlage der Beratungsregelung gibt die amtliche Statistik kaum Aufschluss. Etwa 70 % der Frauen, die 2023 einen Abbruch der Schwangerschaft vornehmen ließen, waren zwischen 18 und 35 Jahre alt. Studien zeigen, dass bei jüngeren Frauen beispielsweise Aspekte wie nichtabgeschlossene Ausbildungen eine Rolle spielen, bei Frauen über 40 Jahren häufiger auch gesundheitliche Bedenken. Ein Schwangerschaftsabbruch hat in den meisten Fällen zwar keine anhaltenden psychischen Folgen, diese können bei bestimmten Risikokonstellationen aber durchaus auftreten. Die Forschung konnte hier inzwischen viele Erkenntnislücken schließen, wie u. a. der in dieses Heft aufgenommene Bericht des Instituts für Qualität und Wirtschaftlichkeit im Gesundheitswesen (IQWiG) zeigt.

Schwangerschaftsabbrüche sind im umfassenderen Kontext der sexuellen und reproduktiven Gesundheit zu sehen. Frauen im gebärfähigen Alter, vor allem auch jüngere Frauen, sollen in ihrer Gesundheitskompetenz bei Themen der sexuellen und reproduktiven Gesundheit unterstützt werden, auch um ungewollte Schwangerschaften zu vermeiden. Sie sollen in der Schwangerschaft selbst eine gute Beratung und Versorgung erfahren, insbesondere soll dies für vulnerable Gruppen und bei Risikoschwangerschaften sichergestellt sein. Die Schwangerschaftskonfliktberatung verfolgt zwar das Ziel des Lebensschutzes, sie soll aber gleichzeitig ergebnisoffen sein und die Autonomie der schwangeren Frauen respektieren. Entscheidet sich eine Frau für einen Abbruch ihrer Schwangerschaft, werden Kosten für eventuelle gesundheitliche Folgen durch die Krankenkassen getragen, der Schwangerschaftsabbruch selbst in der Regel nur bei medizinischer oder kriminologischer Indikation. Die Kosten für einen Schwangerschaftsabbruch liegen nach Angaben von *pro familia*, der Deutschen Gesellschaft für Familienplanung, Sexualpädagogik und Sexualberatung e. V., zwischen 350 € und 600 €. Für Frauen mit geringem oder ohne Einkommen übernimmt das Bundesland, in dem sie leben, auf Antrag die Kosten für den Schwangerschaftsabbruch.

Das vorliegende Heft will Daten zu Einflussfaktoren und Folgen der Entscheidung für einen Schwangerschaftsabbruch berichten sowie Versorgungsfragen beleuchten. Dabei war es ein Anliegen, eine Vielfalt von Themen und die unterschiedlichen Perspektiven der beteiligen Autorinnen und Autoren abzubilden. Einführend werden Eckdaten zu den Schwangerschaftsabbrüchen in Deutschland aus der amtlichen Statistik vorgestellt, internationale Daten und Trends ergänzen das Bild. Mehrere Beiträge widmen sich sozialen, medizinischen und psychologischen Aspekten des Themas – die nicht unabhängig voneinander zu sehen sind.

Den Abschluss dieses Themenheftes bildet ein Beitrag mit ethischen und rechtlichen Überlegungen sowie ein Blick in die Geschichte des Schwangerschaftsabbruchs. Aus medizinisch-ärztlicher Sicht ist insbesondere im Hinblick auf das Ziel der Schwangerschaftsabbruch als solcher eine in der modernen Medizin einzigartige Maßnahme, die mit keiner anderen ärztlichen Handlung zu vergleichen ist. Im Laufe der letzten 150 Jahre lassen sich im Zusammenhang mit dem gesellschaftlichen Wandel auch Perspektivenwechsel auf den Schwangerschaftsabbruch erkennen, von primär religiösen über kriminologische hin zu sozialmedizinischen und sozialpsychologischen Diskursen, was nicht nur die jeweilige politische und rechtliche Regelung des Schwangerschaftsabbruchs und die Wahrnehmung des Themas in bevölkerungsweiten Umfragen bestimmt, sondern auch Voraussetzungen und Leitfragen der Forschung. Damit verbunden ist auch, dass die Perspektive der Frauen eine größere Rolle in den gesellschaftlichen Diskursen und Forschungsansätzen einnimmt.

Gerne hätten wir noch Befunde zur Auswirkung von Schwangerschaftsabbrüchen auf Partner und das familiäre Umfeld mitaufgenommen, da dies auch für die Einbeziehung der Partner in die Entscheidung über einen Abbruch relevant ist. Auch die Studienlage zur besonderen Situation von Migrantinnen aus unterschiedlichen Herkunftskulturen konnte für dieses Heft noch nicht aufbereitet werden, Gleiches gilt für einen internationalen Überblick über Versorgungsfragen und Rechtslagen – Desiderate für spätere Publikationen.

Wir hoffen, den Leserinnen und Lesern trotzdem einen interessanten, vielfältigen und orientierenden Überblick zum besonderen Thema „Schwangerschaftsabbruch in Deutschland“ geben zu können und an einigen Stellen auch Ziele zukünftiger Versorgungsforschung aufzuzeigen.

